# Historical Perspectives and Guidelines for Botulinum Neurotoxin Subtype Nomenclature

**DOI:** 10.3390/toxins9010038

**Published:** 2017-01-18

**Authors:** Michael W. Peck, Theresa J. Smith, Fabrizio Anniballi, John W. Austin, Luca Bano, Marite Bradshaw, Paula Cuervo, Luisa W. Cheng, Yagmur Derman, Brigitte G. Dorner, Audrey Fisher, Karen K. Hill, Suzanne R. Kalb, Hannu Korkeala, Miia Lindström, Florigio Lista, Carolina Lúquez, Christelle Mazuet, Marco Pirazzini, Michel R. Popoff, Ornella Rossetto, Andreas Rummel, Dorothea Sesardic, Bal Ram Singh, Sandra C. Stringer

**Affiliations:** 1 Institute of Food Research, Norwich NR4 7UA, UK; Sandra.Stringer@ifr.ac.uk; 2 Molecular and Translational Sciences Division, United States Army Medical Institute of Infectious Diseases, Fort Detrick, MD 21702, USA; theresa.j.smith.ctr@mail.mil; 3 National Reference Centre for Botulism, Istituto Superiore di Sanita, Rome 299-00161, Italy; fabrizio.anniballi@iss.it; 4 Bureau of Microbial Hazards, Health Canada, Ottawa, ON K1A 0K9, Canada; john.austin@hc-sc.gc.ca; 5 Istituto Zooprofilattico Sperimentale delle Venezie, Treviso 31020, Italy; lbano@izsvenezie.it; 6 Department of Bacteriology, University of Wisconsin, Madison, WI 53706, USA; mbradsha@wisc.edu; 7 Área de Microbiología, Departamento de Patología, Universidad Nacional de Cuyo, Mendoza 450001, Argentina; paulacuervo84@gmail.com; 8 Foodborne Toxin Detection and Prevention Research Unit, Western Regional Research Center, U.S. Department of Agriculture, Albany, CA 94710, USA; luisa.cheng@ars.usda.gov; 9 Department of Food Hygiene and Environmental Health, Faculty of Veterinary Medicine, University of Helsinki, Helsinki 00014, Finland; yagmur.derman@helsinki.fi (Y.D.); hannu.korkeala@helsinki.fi (H.K.); miia.lindstrom@helsinki.fi (M.L.); 10 Robert Koch Institute, Berlin 13353, Germany; dornerb@rki.de; 11 Applied Physics Laboratory, Johns Hopkins University, Baltimore, MD 21218, USA; audrey.fischer@jhuapl.edu; 12 Los Alamos National Laboratories, Los Alamos, NM 87545, USA; khill@lanl.gov; 13 National Center for Environmental Health, Centers for Disease Control and Prevention, Atlanta, GA 30341, USA; skalb@cdc.gov; 14 Army Medical and Veterinary Research Center, Rome 00184, Italy; romano.lista@gmail.com; 15 National Center for Emerging and Zoonotic Infectious Diseases, Centers for Disease Control and Prevention, Atlanta, GA 30329, USA; cluquez@cdc.gov; 16 Institut Pasteur, Bactéries anaérobies et Toxines, Paris 75015, France; christelle.mazuet@pasteur.fr (C.M.); mpopoff@pasteur.fr (M.R.P.); 17 Biomedical Sciences Department, University of Padova, Padova 35131, Italy; marcopiraz@gmail.com (M.P.); ornella.rossetto@unipd.it (O.R.); 18 Institut für Toxikologie, Medizinische Hochschule Hannover, Hannover 30623, Germany; Rummel.Andreas@mh-hannover.de; 19 National Institute for Biological Standards and Control, a Centre of Medicines and Healthcare Products Regulatory Agency, Hertfordshire EN6 3QG, UK; thea.sesardic@nibsc.org; 20 Botulinum Research Center, Institute of Advanced Sciences, Dartmouth, MA 02747, USA; bsingh@inads.org

**Keywords:** botulinum, botulism, neurotoxins, subtypes, *Clostridium botulinum*, guidelines, nomenclature

## Abstract

Botulinum neurotoxins are diverse proteins. They are currently represented by at least seven serotypes and more than 40 subtypes. New clostridial strains that produce novel neurotoxin variants are being identified with increasing frequency, which presents challenges when organizing the nomenclature surrounding these neurotoxins. Worldwide, researchers are faced with the possibility that toxins having identical sequences may be given different designations or novel toxins having unique sequences may be given the same designations on publication. In order to minimize these problems, an ad hoc committee consisting of over 20 researchers in the field of botulinum neurotoxin research was convened to discuss the clarification of the issues involved in botulinum neurotoxin nomenclature. This publication presents a historical overview of the issues and provides guidelines for botulinum neurotoxin subtype nomenclature in the future.

## 1. Historical Perspective of Botulinum Neurotoxin Serotypes

Botulinum neurotoxins (BoNTs) are the most potent naturally-occurring substances, with as little as 50 ng of neurotoxin sufficient to cause human botulism. This minimal lethal dose is estimated from data on the amount of neurotoxin consumed in cases of foodborne botulism and from animal experiments [1,2,3]. Botulinum neurotoxins are 150-kDa proteins that are comprised of a heavy chain (HC-100 kDa) and a light chain (LC-50 kDa). The heavy chains have two functional domains, with the C-terminal domain (H_C_) involved in neurotoxin binding to specific receptors in peripheral nerve terminals (Table 1) and the N-terminal domain (H_N_) involved in translocation of the light chain into the nerve cell cytoplasm [4,5,6]. The light chains are zinc metalloproteases that block the release of the neurotransmitter, acetylcholine, in cholinergic nerves by specific cleavage of SNARE (soluble *N*-ethylmaleimide-sensitive factor (NSF) attachment receptor) proteins (Table 2), leading to flaccid paralysis and botulism [7,8,9]. 

Serological methods were first used to distinguish botulinum neurotoxins more than a century ago. Leuchs [24] showed that botulinum neurotoxins formed by strains of *Clostridium botulinum* isolated following outbreaks of foodborne botulism in Ellezelles (Belgium) and Darmstadt (Germany) were antigenically distinct, with antitoxin raised against one neurotoxin not cross-neutralizing neurotoxin formed by the other strain. Using a similar approach, Burke [25] also recognized two antigenically-distinct botulinum neurotoxins and designated these as serotypes A and B. Strains that formed type A neurotoxin were reported to dominate in the western USA, and strains that formed type B neurotoxin dominated in the eastern USA [26,27]. These pioneering studies established the use of serological methods based on type-specific antitoxins to define and distinguish botulinum neurotoxin serotypes using small animal models. In the decades since Leuchs’ and Burke’s work, the application of the neurotoxin neutralization assay using serotype-specific antisera led to the recognition of seven confirmed botulinum neurotoxin serotypes (types A–G). A potential eighth type (“type H”) was described in 2013. Recent reports have variously described this novel neurotoxin as BoNT/H, BoNT/FA or BoNT/HA [10,28,29,30,31,32,33]. 

Historically, the use of serological methods to identify and characterize botulinum neurotoxins has not been without problems. In 1924 (only a few years after the work of Leuchs and Burke), problems were encountered when serotyping neurotoxin formed by newly-identified BoNT/C strains that were tested using specific antisera. It was found that antisera produced from type C strains that were isolated from fly larvae and chickens were able to neutralize type C neurotoxin from several strains isolated in the USA, as well as the “Seddon” type C strain that was isolated from cattle with botulism in Australia, but that antisera produced from the “Seddon” strain neutralized only its homologous toxin [34]. It is now known that these “type C” toxins are BoNT/CD chimeras composed of the 2/3 type C and 1/3 type D sequence, whereas true type C1 toxins are produced by Seddon-like strains [35].

Similar issues arose in the serotyping of toxins from multiple type A and type F strains in Argentina [36]. Significant differences in the efficiency of neutralization were noted, particularly among the BoNT/F toxins. These observations led the researchers to conclude, “there is a general tendency to accept the antigenic homogeneity of the botulinum toxins within each type, and from the year 1924 in which the serological relations among the type C strains were described, later denominated type Cα and Cβ (currently CD and C1), up to this date it was never known with precision what to do with these strains, from the point of view of their classification. But the biologic reality is that there are serologic variations of differing magnitude in strains within each type, evidenced by significant differences in antitoxin consumption in cross neutralization, being the most obvious cases those observed in the strains of type C and F” [37].

These observations pointed to a level of intratypic serological diversity that underlies the “serotype” designations. It was recognized that these differences may have an impact on the effectiveness of botulism treatment, as antitoxins have been raised against a single toxin subtype per serotype. For example, all currently-produced commercial botulinum antitoxins were produced following vaccination with BoNT/A1, BoNT/B1 and either BoNT/E1 or BoNT/E3 toxoids. The few research studies that have been published evaluating the effectiveness of such antisera have shown differential protection against the spectrum of toxins within a single serotype [38,39]. In addition, the impact of intratypic serological diversity on the effectiveness of current antitoxin treatments remains largely unknown.

## 2. Historical Perspective of Botulinum Neurotoxin Subtypes

Early work had suggested that each strain of *C. botulinum* formed a single type of botulinum neurotoxin. However, using mouse tests and specific antisera, Giménez and Ciccarelli described a strain that formed two distinct types of neurotoxin, with a major amount of type A toxin activity and a minor amount of type F toxin activity [40]. This strain was designated type A, subtype Af [40]. A number of strains are now described that form more than one type of botulinum neurotoxin [37,41,42]. For more than a decade now, however, the term subtype has been used in a different way, that is to describe intratypic neurotoxin variation based on the amino acid sequence of the neurotoxin (derived following sequencing of the neurotoxin gene). A numerical notation has also been introduced, so that the subtypes are designated BoNT/A1, BoNT/A2, BoNT/A3, etc. [43,44,45,46,47,48,49] (Table 3).

The development of techniques to enable the sequencing of individual genes has substantially increased our understanding of botulinum neurotoxin diversity. Within a three-year time span (1990–1993), sequences representing one member from each of the seven neurotoxin serotypes became available [50,51,52,53,54,55,56,57]. Five years after initial sequences for each serotype were made public, sequences for eight alternative neurotoxin subtypes had been published [35,58,59,60,61,62,63]. Five of these subtypes were known to differ in some way from the “reference” toxins for each serotype prior to sequencing. This included a strain that produced a type A1 toxin and contained a nonfunctional BoNT/B gene (an A1(B) strain) [64]. 

The first *C. botulinum* whole genome sequence was published in 2007 [47], and many full genomes in addition to individual neurotoxin-encoding genes have now been sequenced. Sequencing has confirmed the distinctiveness of the seven botulinum neurotoxin serotypes (types A–G), with amino acid differences between the seven neurotoxin serotypes ranging from 37.2%–69.6% [65] (Table 4). Furthermore, studies of the functionality of the botulinum neurotoxins also support the classification of seven serotypes. Botulinum neurotoxin light chains possess endopeptidase activity and selectively cleave proteins of the neurotransmitter vesicle docking/fusion complex, preventing the formation of a stable complex [8,9]. BoNT/A, C and E cleave SNAP-25 at distinct sites; BoNT/B, D, F and G cleave VAMP-1/2/3 at distinct sites; BoNT/C can also cleave syntaxin 1A/C (Table 2). Each neurotoxin subtype within a serotype cleaves its target substrate at the same single conserved peptide bond, except for BoNT/F5 [23] (Table 2).

The technology resulting in the production of monoclonal antibodies provided a new way of evaluating these toxins serologically. Monoclonal antibodies developed against BoNT/A [66], BoNT/B [67], BoNT/C1 [68] and BoNT/E [69] were used to develop new toxin detection assays and also to discover new aspects of toxin structure and activity. However, these antibodies have been of limited use as predictors of subtype-level differences, since monoclonal antibody epitopes are, at most, limited to 5–7 continuous or discontinuous amino acids. Many toxin subtypes are very closely related, so that most monoclonal antibodies will bind multiple toxin subtypes, severely limiting their discriminating power and making them unsuitable for toxin subtype determinations.

**Figure 1:**
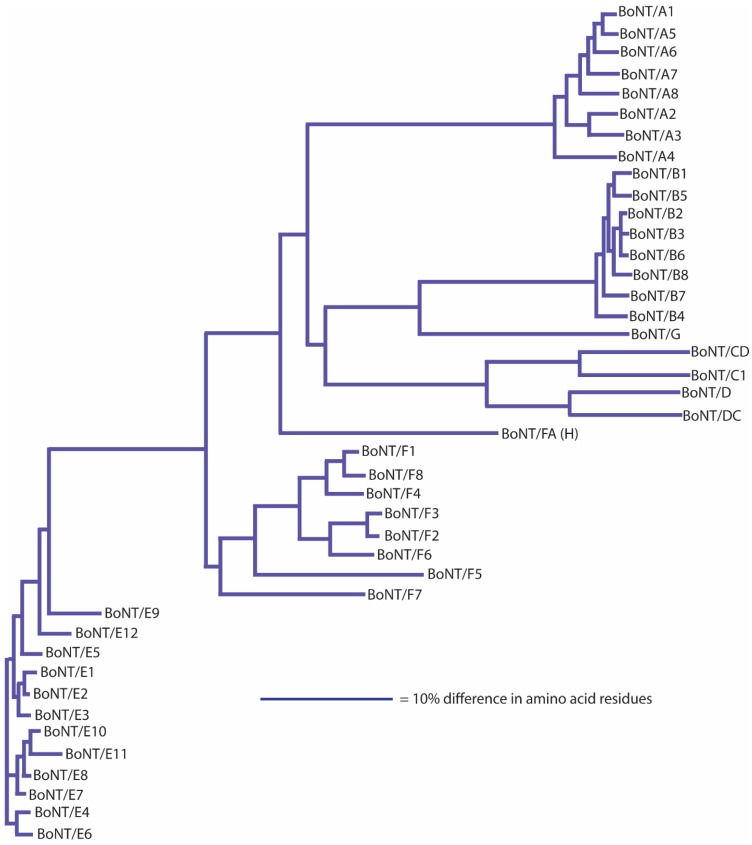
Dendrogram showing the relationship of all published/publicly-posted BoNT subtypes. The dendrograms were generated from protein sequence data using ClustalW with the representatives listed in Table 3. BoNT/FA is also known as BoNT/H and BoNT/HA (see the text for further details).

Initial studies using monoclonal antibodies, however, often reported that the antibodies would neutralize some, but not all, subtypes of a specific serotype, suggesting variability within each neurotoxin serotype [45,69,70]. The extent of this variability is now being revealed through sequencing of whole genomes and individual neurotoxin-encoding genes. A landmark article on sequence variation among botulinum neurotoxin serotypes published in 2005 described within-serotype variations among neurotoxin sequences as being of two types: those that were virtually identical and those that were more variable and differed by at least 2.6% in amino acid sequence. This observation was based on a study of 49 neurotoxin sequences (each serotype, with the exception of type G, was represented by 4–17 sequences) [45]. Subsequent studies sorted these and additional toxin gene sequences into differing phylogenetic clades, which were then identified as new subtypes [71,72]. The identification of novel subtypes has thereby been based primarily on the sequence of the botulinum neurotoxin gene and/or derived amino acid sequence. There are more than forty neurotoxin subtypes presently described in the literature (Table 3; Figure 1). 

**Figure 2:**
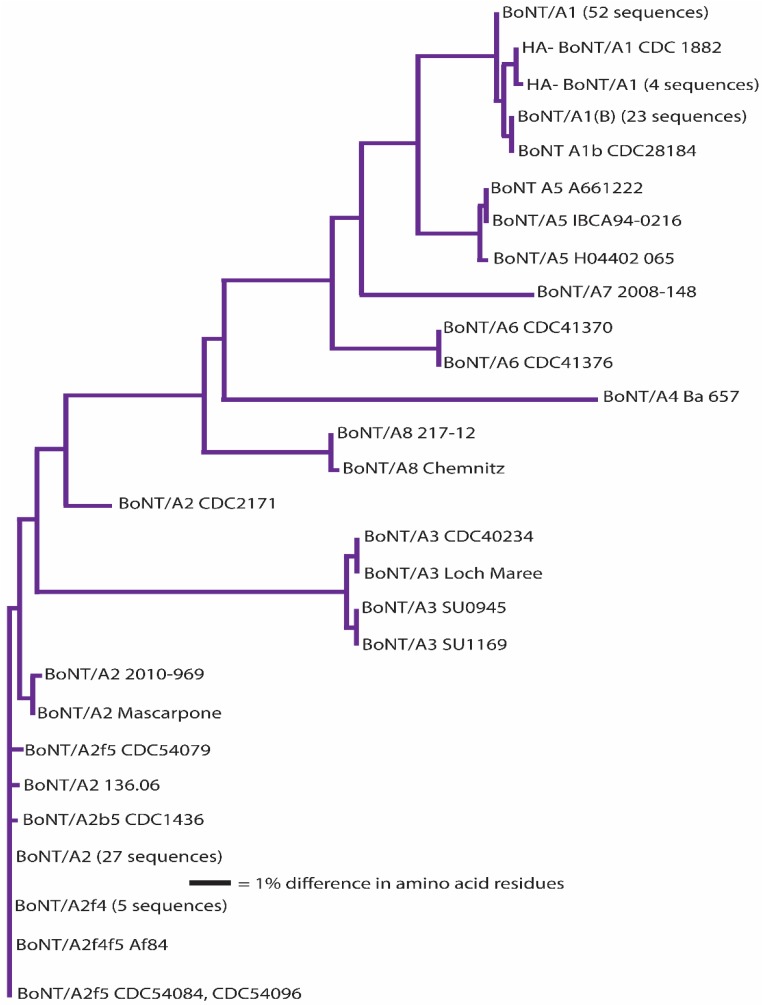
Dendrogram showing the relationships of BoNT/A subtypes. A total of 127 amino acid sequences were analyzed. “HA-“ indicates BoNT/A1 encoding gene within a toxin cluster lacking genes encoding hemagglutinin proteins.

**Figure 3:**
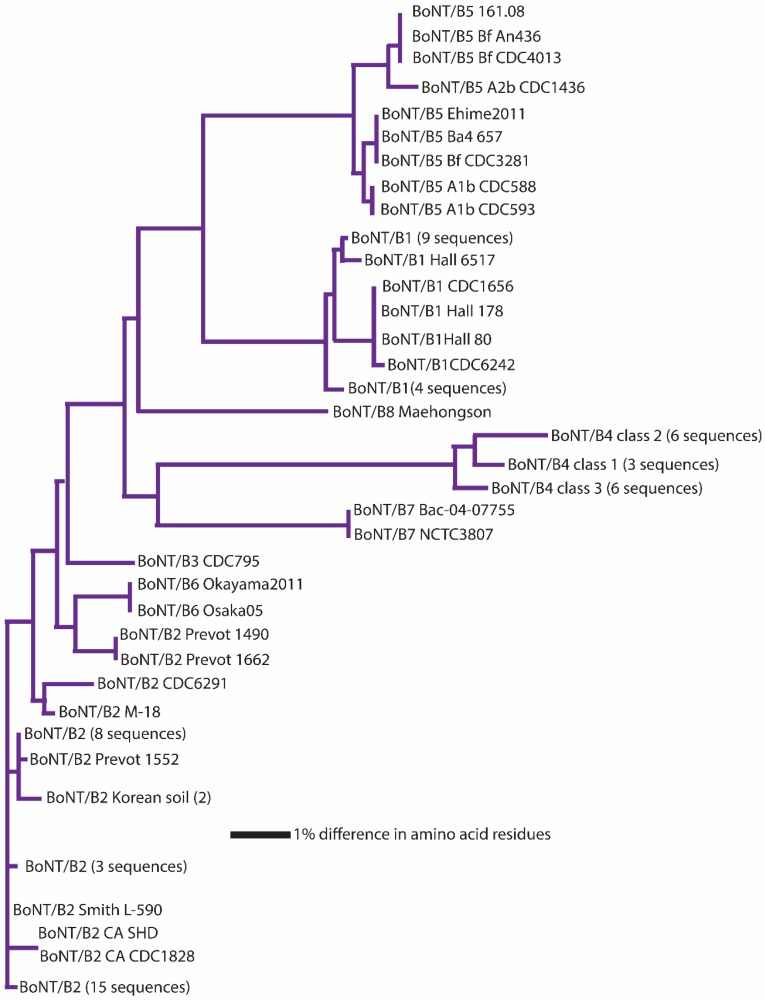
Dendrogram showing the relationships of BoNT/B subtypes. A total of 91 amino acid sequences were analyzed. The BoNT/B subtypes show the closest relationships, with amino acid differences ranging from 1.6%–7.1%.

**Figure 4:**
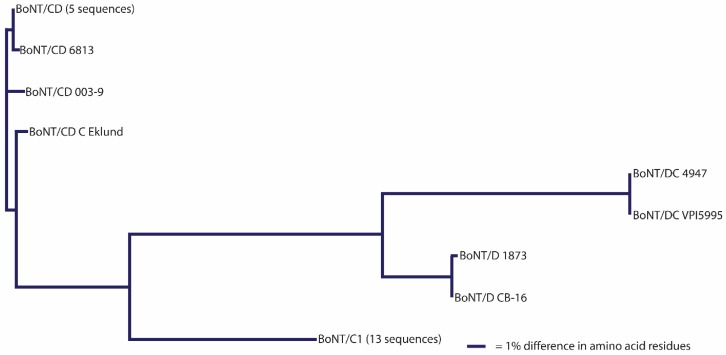
Dendrogram showing the relationships of BoNT/C and BoNT/D subtypes. A total of 23 amino acid sequences were analyzed. The mosaic nature of these subtypes results in large differences in amino acid sequence (23.5%–48.8%).

**Figure 5:**
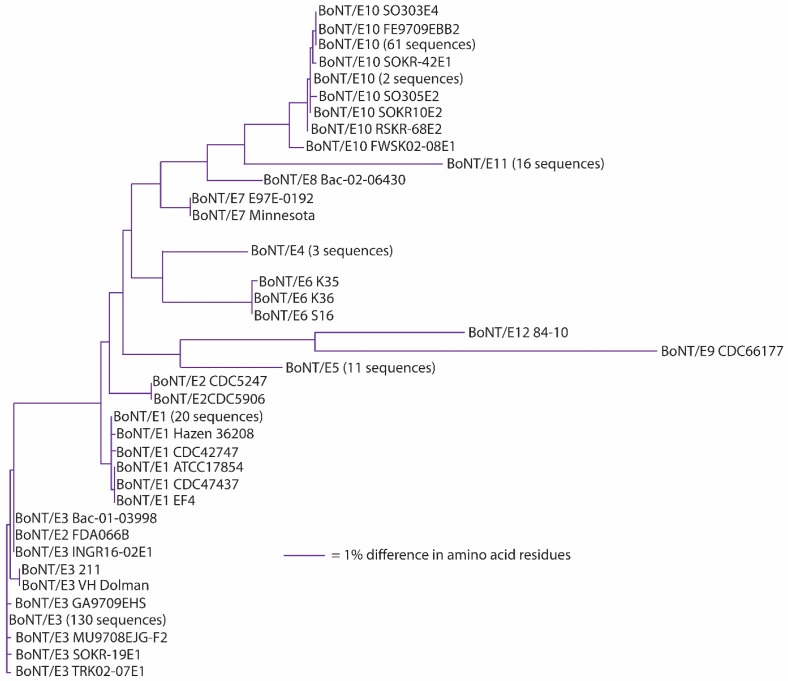
Dendrogram showing the relationships of BoNT/E subtypes. A total of 235 amino acid sequences were analyzed.

**Figure 6:**
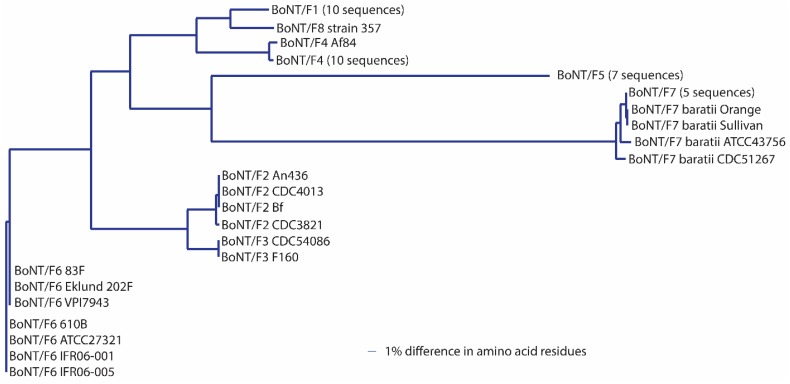
Dendrogram showing the relationships of BoNT/F subtypes. A total of 52 amino acid sequences were analyzed. The scale for this dendrogram is significantly smaller than with the others due to the wider range of identity differences (7.8%–36.2%) within this serotype.

It has been suggested that a *C. botulinum* neurotoxin could be defined as a distinct subtype if it encoded a protein sequence that differed from the prototype neurotoxin by at least 2.6% [46,72,73,74,75,76,77,78,79,80,81,82,83]. A comparison of neurotoxin sequences from 127 BoNT/A-, 91 BoNT/B-, 23 BoNT/C- and BoNT/D-, 235 BoNT/E- and 50 BoNT/F-producing strains obtained from various sources has been conducted; the results are reported in Table 3, Table 4, Table 5, Table 6, Table 7, Table 8 and Table 9. This includes published and unpublished neurotoxin sequences, many of which were identical. While no particular efforts to publicly post the redundant sequences were made, examples of each subtype, including strain name, source and GenBank accession number, are listed in Table 3. Figure 1, Figure 2, Figure 3, Figure 4, Figure 5 and Figure 6 illustrate the range of neurotoxin sequence diversity among these strains. While most presently-described neurotoxin subtypes differ from each other by more than 2.6% at the amino acid level, some BoNT/B subtypes and BoNT/E subtypes do not meet this criterion (Table 6 and Table 8). Additionally, it is more common for strains of *C. botulinum* Group III to form a chimeric or hybrid protein that combines domains of BoNT/C and BoNT/D neurotoxin, rather than a distinct BoNT/C or BoNT/D neurotoxin (Table 7; Figure 4).

Discussions as to the relationship between sequence differences and potential immunological or functional differences have led to studies comparing toxin characteristics versus sequence. The majority of these studies involved differential binding to antibodies. As expected, binding differences were more noticeable when monoclonal antibodies were used, but quantitative differences were also noted with assays involving polyclonal antibodies [38,45]. However, as noted above, these immunological differences cannot reliably identify distinctive toxin subtypes. 

Biological and functional activities are presumed to be largely conserved within individual neurotoxin serotypes. Currently, the only naturally-occurring amino acid sequence changes that have led to functional differences were reported with subtype BoNT/F5, whose enzymatic domain differs from all other BoNT/F enzymatic domains by greater than 50% in amino acid sequence (Table 2), leading to differences in the enzymatic target site [23]. All other subtypes within a serotype utilize the same enzymatic cleavage target substrates and sites. 

Even though studies of toxin:receptor interactions of subtypes are limited, current information indicates that all toxin subtypes within a serotype also interact with the same receptor targets (Table 1), with one exception. BoNT/DC interacts with synaptotagmin 1 and 2; the putative receptor for BoNT/D is *N*-glycosylated SV2A and B; and BoNT/C appears to interact solely with gangliosides [15,16,17]. This difference may not be too surprising, as BoNT/DC is a hybrid toxin with a BoNT/C-like receptor-binding domain that differs in amino acid sequence from BoNT/D by 60.2% and from BoNT/C by 22.2%. These differences have directed different receptor interactions for each of the toxins. It should also be noted that subtype quantitative binding differences to receptors were seen with two BoNT/B subtypes. BoNT/B1 and BoNT/B2 both interact with synaptotagmin, but BoNT/B1 binds both synaptotagmin 1 and 2, with binding affinities of 3.4 and 0.52 nM, respectively, while BoNT/B2 binds only synaptotagmin 2, with an intermediate binding affinity of 2.4 nM [44]. There is a significant amino acid sequence difference (8.1%) in the receptor binding domains of BoNT/B1 and BoNT/B2. BoNT/A8 has also been reported to have a reduced affinity to ganglioside receptors compared to BoNT/A1 [81]. Additional differences in catalytic activity have also been described among BoNT/A subtypes [84,85,86,87,88].

It is presently not feasible to use biological, structural, immunological or functional characteristics to subtype botulinum neurotoxins, as knowledge is limited. However, a sequencing-based approach can be used to rapidly categorize botulinum neurotoxin subtypes, to avoid confusion in the literature and to facilitate future research endeavors. One important benefit of this approach is that it allows for comparison of neurotoxins formed by strains located in different laboratories. 

When analyzing presently-published toxin subtypes, in which subtype categorization has been based on amino acid sequence differences, it is important to recognize that each serotype shows a unique pattern of between-subtype and within-subtype differences (Table 5, Table 6, Table 7, Table 8 and Table 9). In the case of BoNT/A, inter-subtype differences range from 2.9%–15.6% (Table 5; Figure 2). Intra-subtype differences are much smaller (≤0.8%, with one exception), thereby providing a sufficient margin of discrimination in sequences that are within subtypes versus those between subtypes. The exception is strain CDC 2171, which differs from other BoNT/A2 by ~2.5%. Due to an apparent recombination event, this toxin shares 100% identity with BoNT/A2 Kyoto-F for approximately 2/3 of the sequence, but differs by 5.6% in the terminal H_C_ region of the molecule. The margin of discrimination among BoNT/A subtypes is similar to the scenario with most BoNT/F subtypes, where inter-subtype differences range from 3.0%–36.2%. Intra-subtype differences are ≤0.6% with the exception of BoNT/F7, which shows an intra-subtype difference of 1.7% among the eight sequences that were analyzed (Table 9). Removal of a single BoNT/F7 sequence, from strain ATCC 43756, results in a reduction in variability within this subtype from 1.7% to 0.8%. 

BoNT/E subtype sequences, with the exception of BoNT/E9 and BoNT/E12, are closely related (Figure 5). Amino acid differences among BoNT/E1-BoNT/E8 range from 0.9%–5.9%, but intra-subtype differences of 0.1%–0.2% are seen within BoNT/E1, BoNT/E2, BoNT/E4-6 and BoNT/E11 subtypes (Table 8). The lone BoNT/E9 strain sequence differs from BoNT/E1-8, BoNT/E10 and BoNT/E11 by 10.1%–11.8% in amino acid residues, making it distinctive among the BoNT/E subtypes [78]. One issue that has arisen when distinguishing different subtypes having limited differences has been the use of phylogenetic clade analysis of neurotoxin nucleotide sequences, instead of comparisons of their amino acid differences, as the basis for discrimination [72,89]. While BoNT/E1, BoNT/E2 and BoNT/E3 clearly sort into distinct, but closely-related, phylogenetic clades [89,90], amino acid analysis of their sequences shows their differences range from 0.9%–1.8% when BoNT/E2 and BoNT/E3 are compared to BoNT/E1 (Table 8). If the 2.6% amino acid difference guideline had been applied, this would be considered a single larger subtype. While it has been decided that historical subtype designations will continue to be used (see below), it is useful to understand that BoNT/E1, BoNT/E2 and BoNT/E3 could be considered as a single subtype entity. The effect of analyzing large numbers of sequences is seen with BoNT/E3 (toxin sequences from 143 strains) and BoNT/E10 (toxins sequences from 36 strains), where intra-subtype sequence differences were 0.6% and 0.8%, respectively. It is possible that as additional toxin sequences become available, intra-subtype differences may increase. 

BoNT/B shows the greatest degree of intra-subtype variability of any serotype (Figure 3). Inter-subtype differences range from 1.6%–7.1%, and intra-subtype differences range from 0.8%–2.1%. A clear relationship can be seen among BoNT/B2, BoNT/B3 and BoNT/B6 (within-subtype amino acid differences of 1.6%–1.9%), which is similar to the situation with BoNT/E1, BoNT/E2 and BoNT/E3. BoNT/B2, BoNT/B3 and BoNT/B6 were also initially differentiated using phylogenetic clade analysis, not amino acid differences [72,73], with the result that BoNT/B2, BoNT/B3 and BoNT/B6, like the BoNT/E1/E2/E3 grouping, could be considered a single subtype. In addition, with BoNT/B, the within-subtype variability is higher overall than with other toxin types, ranging from 0.8%–1.9%. The BoNT/B2 and BoNT/B4 subtypes are particularly variable (Table 6). This unique ranging of BoNT/B2 and related subtypes may indicate that horizontal genetic interactions between certain BoNT/B-producing strains show a higher level of activity than that seen among other serotypes. 

These recombination events may be the major factor responsible for the proliferation of subtypes seen within this toxin. It is important to note these events as part of toxin characterizations; however, attempts to define the toxin subtypes on the basis of these events could become challenging. A prime example is the current dispute over the nomenclature for the newly-described novel toxin known as BoNT/H, BoNT/HA or BoNT/FA, depending on how it is characterized [10,28,29,30,31,32,33].

An interesting finding is the identification of a novel homolog of BoNT in the genome of a non-*Clostridium* species [91]. The homolog, named BoNT/Wo to correspond with its bacterial host (*Weissella oryzae*), was verified by the Montecucco laboratory to have BoNT-like enzymatic activity [92]. However, at the classification level, more work is required to determine whether BoNT/Wo should be considered a new family altogether or a highly divergent member of the BoNT family.

## 3. Historical Perspective of Botulinum Neurotoxin Forming Clostridia

Historically, the production of a botulinum neurotoxin was the only criterion for the species nomenclature for these strains, so that all botulinum neurotoxin-producing clostridia were known as *Clostridium botulinum*. Today, at least six physiologically- and genetically-distinct bacteria are known to form botulinum neurotoxins [47,65,93,94,95,96,97]. Currently-recognized species include *C. botulinum* Groups I–IV, some strains of *C. baratii*, *C. butyricum* [98] and possibly also neurotoxin-producing *C. sporogenes*. *C. botulinum* comprises four discrete groups of bacteria. *C. botulinum* Group I (proteolytic *C. botulinum*) strains are mesophilic and form spores of high heat resistance [94]. *C. botulinum* Group I strains produce BoNT/A, BoNT/B, many of which were identical, and/or BoNT/F. The number of neurotoxin genes located in the Group I genomes and the number of neurotoxins produced is variable, with strains possessing up to three neurotoxin genes, and producing one or, more rarely, two or three distinct neurotoxins [93]. Nontoxic representatives have also been isolated. *C. botulinum* Group I strains are primarily responsible for human botulism. *C. botulinum* Group II (non-proteolytic *C. botulinum*) strains are psychrotrophic and form spores of moderate heat resistance [94]. *C. botulinum* Group II strains produce either BoNT/B4, BoNT/E or BoNT/F6. Group II strains are not known to produce multiple toxins, however sequencing of the genomes of *C. botulinum* Group II BoNT/F6 strains revealed that they also contain fragments of a type B and a type E neurotoxin gene [99]. Non-toxic strains have been described [100]. *C. botulinum* Group II causes human botulism. Neurotoxin encoding genes of *C. botulinum* Groups I and II are located on the chromosome or on a plasmid [3,42,49,65,75,94,95,101,102,103,104]. *C. botulinum* Group III strains, also included in *C. novyi sensu lato* [96], are mesophiles and cause botulism in various animal species. Strains form BoNT/C or BoNT/D, although more frequently a hybrid BoNT/CD or BoNT/DC neurotoxin is produced [35,105]. *C. botulinum* Group IV (also known as *C. argentinense*) strains form BoNT/G, which has not been definitively associated with human or animal botulism [106]. Some strains of *C. baratii* form type F7 neurotoxin, and some strains of *C. butyricum* form type E4 or E5 neurotoxin; both bacteria are associated with human botulism [98]. It has also recently been noted that some BoNT/B-producing strains formerly thought to be within *C. botulinum* Group I may be neurotoxigenic strains of *C. sporogenes* [49,107,108].

The earliest botulinum neurotoxins described were a type B neurotoxin formed by a strain of *C. botulinum* Group II and a type A neurotoxin formed by a *C. botulinum* Group I strain [109,110]. However, one important difference between these neurotoxins was not due to characteristics of the neurotoxins, but rather to characteristics imparted by the bacteria themselves. The neurotoxin is formed as a progenitor toxin, a single 150-kDa polypeptide. Strains of *C. botulinum* Group I produce proteolytic enzymes that are responsible for post-expression processing of the neurotoxin, leading to a more active di-chain structure with a 50-kDa light chain attached to a 100-kDa heavy chain by a disulfide bond [111]. Strains of *C. botulinum* Group II lack these enzymes, and the neurotoxin remains as a single polypeptide chain to be fully activated by host proteases [112].

## 4. Developing a Way Forward with Regard to a Nomenclature for Botulinum Neurotoxin Subtypes

While the identification of neurotoxin serotypes and subtypes has aided in understanding the epidemiology of neurotoxin-producing clostridia and in the development and screening of effective diagnostics and treatments for botulism, the increasing numbers of toxin subtypes that are being identified has posed a challenge for researchers. New toxin variants are constantly being discovered, and there is confusion as to the range of variation within each subtype. It is not always apparent whether a “new toxin” should be described as a new “subtype” or not and what is the correct designation for this neurotoxin. For example, multiple laboratories may be publishing the same neurotoxin subtypes as different designations without the knowledge that they are related, or identical neurotoxin subtypes may be identified as a particular neurotoxin subtype in one publication and as another neurotoxin subtype in a different publication. The nomenclature picture is confusing, and a systematic approach to neurotoxin subtype nomenclature is urgently needed.

To address this issue, a committee was formed to consider the problem and propose solutions. Initial efforts involved a survey of researchers working on various aspects of botulinum neurotoxin research. Seventy-eight responses were received.

- The majority felt that nomenclature standardization was somewhat or very important
- The majority preferred the term “toxin subtype” to best describe within-serotype toxin differences
- The highest importance was given to nucleotide or amino acid differences; however, half of the responders felt that the nomenclature should be also be based on functional differences.

A group of more than twenty researchers from North and South America and Europe then volunteered to participate in the drafting of guidelines designed to aid researchers with neurotoxin subtype nomenclature. In addition, the feasibility that a database could be set up to analyze submitted toxin sequences and determine for the researcher if their neurotoxin is a new or existing subtype was investigated. A second questionnaire was sent to each volunteer to further clarify the consensus opinions for nomenclature guidelines. There was agreement on several issues:

(1) The best term to describe within-serotype differences was “subtype”.
(2) Subtype discrimination should be based on protein sequences derived from sequencing of the encoding gene, which can be obtained quickly and shared among the botulinum research community as a whole.
(3) Previously-published subtypes should be maintained as identified, with adjustments being made only to avoid confusion.
(4) There was a need for a specialized screening system to aid in organizing subtype nomenclature, and new sequences should be submitted to public databases as soon as practically possible.

## 5. The Proposed Way Forward with Regard to a Nomenclature for Botulinum Neurotoxin Subtypes

The unique characteristics of the different subtypes within each serotype and their relationships with each other make the selection of a single standard problematic. However, the objective here is to provide a level of organization in nomenclature, not to provide detailed, exacting categorization of each new toxin that is discovered. The majority of the approximately forty botulinum neurotoxin subtypes presently described in the literature was based on the amino acid sequence of the proteins, derived from the encoding gene. A study of 49 neurotoxin sequences, published in 2005, reported that subtypes differed by at least 2.6% in amino acid sequence [45]. Although this is a relatively arbitrary cut-off, it has provided the basis for most genetic subtype designations for the past decade. More than 500 neurotoxin sequences were recently compared, and 41 distinct subtypes have been identified (Table 5, Table 6, Table 7, Table 8 and Table 9). Although most of the described subtypes differed by more than 2.6% at the amino acid level, some BoNT/B and BoNT/E subtypes did not [113]. 

This raises the question as to whether: (i) the present subtypes should be accepted; or (ii) a cut-off of a 2.6% difference should be rigidly applied and some neurotoxin subtypes re-designated. There is also the issue of how subtypes should be identified in the future. The committee proposes that:

(1) Subtypes should be determined from the amino acid sequence derived by gene sequencing.
(2) Current subtypes named in the literature will be retained (except where either two distinct neurotoxins are given the same subtype or one neurotoxin is known as two subtypes) (Table 3). This would include retention of subtypes BoNT/B2, BoNT/B3 and BoNT/B6, which differ by 1.6%–1.9%; BoNT/E1 with BoNT/E2 or BoNT/E3, which differ by 1.0%–1.8%; and BoNT/E1 with BoNT/E7 or BoNT/E8, which differ by 1.8%–2.2%.
(3) All future designated subtypes must differ from all known subtypes by more than 2.6% at the amino acid level, and to avoid future confusion, a centralized procedure will be used to aid in assigning appropriate subtype designations to these toxins.
(4) As this nomenclature is based on the protein sequence derived following sequencing of the encoding gene, it is proposed that the term “subtype” or “genetic subtype” be used to distinguish from nomenclature based on serotyping alone (as in “subserotype”).
(5) All publications on BoNTs should disclose not only the serotype and subtype designations of the toxin, but also the strain it is derived from, and the source of the strain. The toxin sequences should also be publically posted (e.g., GenBank) and the accession number given.

It is recognized that this scheme considers all amino acid changes as equivalent and that some changes will be more significant than others. Furthermore this scheme should not be construed to predict biological function, structure or reflect neurotoxin evolution, but is rather a way of broadly categorizing related neurotoxin sequences and perhaps allowing investigators to target specific sequences for further study. 

Additionally, it is apparent that some of the present neurotoxin subtypes are a hybrid of other subtypes. For example, BoNT/A2 is a hybrid of BoNT/A1 and BoNT/A3 [72], and BoNT/F6 is a hybrid of BoNT/F1 and BoNT/F2 [95]. The designation of these hybrids as distinct subtypes is supported, but it should be noted in manuscripts that these are hybrids. It is recognized that a majority of BoNT/C and BoNT/D are hybrids, which are not given numeric appellations, but are reported as BoNT/CD or BoNT/DC chimeric toxins [35]. Two exotoxins expressed simultaneously with BoNT/C and/or BoNT/D, which are not neurotoxins, have been designated as type C2 and C3 toxins [113]. In order to avoid confusion with the hybrid botulinum neurotoxins and the C2 and C3 toxins, the terms BoNT/CD and BoNT/DC will continue to be used to designate hybrid BoNT/C and D subtypes.

## 6. Development of a Screening System for Preliminary Identification of Novel Botulinum Neurotoxin Subtypes

The system for clarification of toxin subtype would function as follows:

(1) Prior to publication, a researcher would determine the neurotoxin amino acid sequence following sequencing of the encoding gene and compare it to known subtype sequences.
(2) If the new sequence differs from all known subtypes by more than 2.6% at the amino acid level, a new subtype designator would be needed.
(3) To request a subtype designator, the researcher would submit a table showing amino acid percent differences of the new subtype with representative sequences of known subtypes to the Centers for Disease Control and Prevention, Atlanta, Georgia, USA (CDC) (bontsubtype@cdc.gov). It is not necessary to submit the actual nucleotide or amino acid sequence.
(4) A new subtype designator would be relayed to the researcher for publication and reserved for a defined period of time.
(5) It is possible that two or more laboratories request new subtype designations for the same neurotoxin serotype at around the same time, previous to publication. To avoid these laboratories publishing the same neurotoxin subtype with different designations or different neurotoxin subtypes with the same designator, the CDC would make both labs aware of the potential conflict. The laboratories would be responsible for communicating to each other to compare sequences and ensure that the subtyping nomenclature is correct.

The actual toxin sequence would remain with the researcher throughout the procedure until it is published. This ensures control of the data while clarifying its classification. It is stressed, however, that the toxin sequences should be publically posted (e.g., GenBank) and published as soon as possible and that publications should clearly state the strain and its source, its subtype and the deposited sequence accession number. 

## 7. Summary

For several decades, the standard method for identifying and characterizing botulinum neurotoxins has involved animal tests using serotype-specific antisera, with the range of variation in response to these toxins occasionally providing challenges as to the assignment of specific serotypes. In addition, the reagents necessary for these procedures are becoming scarce, and there is a strong desire to minimize the use of experimental animals. The ability to sequence neurotoxin genes and derive the associated neurotoxin protein sequence has become widespread over the past 25 years and has revealed not only details concerning serotype and subtypes, but also underlying variation that might not be noticed when using serotyping antisera. The proliferation of inexpensive, rapid sequencing methods has enabled laboratories worldwide to characterize neurotoxins in this consistent way. We note for future consideration that there is a developing interest in the potential of classifying botulinum neurotoxins based on their enzymatic activity (including substrate cleavage patterns). 

We propose to take advantage of sequencing methods to categorize botulinum neurotoxin subtypes and to clarify subtype nomenclature through a screening system of new neurotoxin sequences that will eliminate uncertainties as to the nomenclature of these toxins. A database will be used to compare neurotoxin sequence differences with known toxin subtypes and provide guidelines as to whether the newly-submitted sequences are related to known toxin subtypes or whether they represent novel subtypes that can be published as such with confidence.

## Figures and Tables

**Table 1:** Synaptic vesicle proteins that act as receptors for botulinum neurotoxins. BoNT, botulinum neurotoxin.

Serotype	Protein Receptor	Binding Site	References
BoNT/A	*N*-glycosylated SV2A, B, C	H_CN_-H_CC_	[10,11,12]
BoNT/B	Synaptotagmin I and II	H_CC_	[13,14]
BoNT/C	----- *		[15]
BoNT/D	*N*-glycosylated SV2A, B, C		[16]
BoNT/DC	Synaptotagmin I and II	H_CC_	[17]
BoNT/E	N-glycosylated SV2A, B	H_CN_-H_CC_	[18,19]
BoNT/F	*N*-glycosylated SV2A, B, C		[20,21]
BoNT/G	Synaptotagmin I and II	H_CC_	[14,22]

* BoNT/C interacts with ganglioside only; there is no protein receptor identified so far.

**Table 2:**
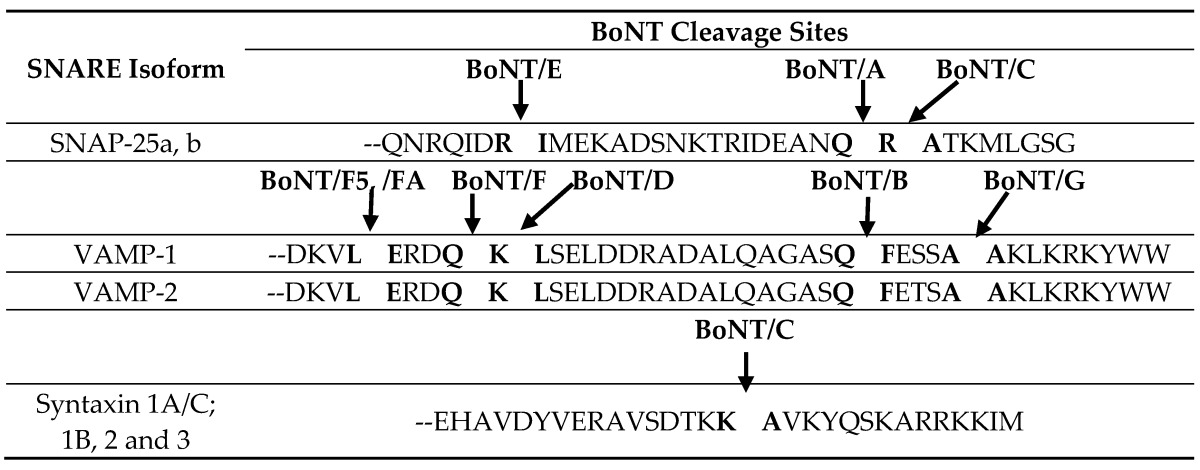
The enzymatic targets and cleavage sites of various botulinum neurotoxins. The table is a modification of [7] with additional data from [23].

**Table 3:** Representative strains of BoNT subtypes. Note that these prototype strains were used to produce the comparisons in Table 4, Table 5, Table 6, Table 7, Table 8 and Table 9.

Serotype	Subtype	Representative Strain	Source/Date	Sequence Accession #
BoNT/A	A1	ATCC 3502	peas/California, 1922	CAL82360
	A2	Kyoto-F	infant botulism/Japan, 1978	CAA51824
	A3	Loch Maree	duck paste/Scotland, 1922	ACA57525
	A4	Ba657	infant botulism/Texas, 1976	ACQ51417
	A5	H04402 065	wound botulism/U.K., 2004	ACG50065
	A6	CDC 41370	food/Mexico, 1996	ACW83608
	A7	2008-148	enchiladas/France, 2008	AFV13854
	A8	Chemnitz	green bean salad/Germany, 2007	AJA05787
BoNT/B	B1	okra	okra/Tennessee, 1939	ACA46990
	B2	111	infant botulism/Japan, 1995	BAC22064
	B3	CDC 795	Unknown	ABM73977
	B4	Eklund 17B	marine sediments/Pacific coast, 1965	ABM73987
	B5	Ba657	infant botulism/Texas, 1976	ACQ51206
	B6	Osaka05	infant botulism/Japan, 2005	BAF91946
	B7	Bac-04-07755	infant botulism/New York, 2004	AFD33678
	B8	Maehongson	foodborne botulism/Thailand, 2010	AFN61309
BoNT/C	C1	Stockholm	mink/Sweden	BAA14235
	CD	6813	soil/Maryland	BAA08418
BoNT/D	D	1873	ham/Chad, 1958	EES90380
	DC	VPI 5995	South Africa	ABP48747
BoNT/E	E1	Beluga	whale/Alaska, 1952	CAA43999
	E2	CDC 5247	Unknown	EF028404
	E3	Alaska E43	Alaska	ABM73980
	E4	BL5262	infant botulism/Italy, 1984	BAC05434
	E5	LCL155	soybean-wax gourd paste/China	AB037704
	E6	K35	fish/Finland, Baltic Sea	CAM91125
	E7	IBCA97-0192	whitefish/California, 1997	AER11391
	E8	Bac-02-06430	round goby/Lake Erie, 2002	AER11392
	E9	CDC 66177	environmental/Argentina, 1995	AFV91339
	E10	FWKR11E1	freshwater/Canada, 2004	KF861920
	E11	SW280E	seawater/Canada, 2001	KF861879
	E12	84-10	ham/France, 2009	KF929215
BoNT/F	F1	Langeland	duck paste/Denmark, 1958	ABS41202
	F2	CDC 3281	infant botulism/Texas, 1982	CAA73972
	F3	VPI4257 (F160)	soil/Argentina, ~1968	ADA79575
	F4	CDC54089	anchovies/Argentina, 1984	GU213221
	F5	CDC54075	soil/Argentina, 1978	GU213212
	F6	Eklund 202F	marine sediments/Pacific coast, 1965	AAA23263
	F7	Sullivan	adult botulism/New York, 2007	ADK48765
	F8	I357	asparagus/Italy, 2005	AUCZ00000000
BoNT/G		CDC 2741	autopsy specimen/Switzerland, 1978	KIE44899
BoNT/FA (H) *		CFSAN024410 (IBCA 10-7060)	infant botulism, 2010	KGO15617

* This neurotoxin is variously described as BoNT/FA, BoNT/H and BoNT/HA (see the text).

**Table 4:** Amino acid differences among BoNT serotypes *.

Serotype	A	B	C	D	E	F	G
A	-----	62.5%	69.6%	68.8%	62.3%	61.3%	62.0%
B		-----	69.2%	67.4%	64.1%	62.6%	**42.9%**
C			**-----**	**48.6%**	69.1%	69.1%	67.5%
D				-----	68.4%	67.3%	66.0%
E					-----	**37.2%**	63.6%
F						-----	63.2%
G							-----

* Data for subtypes A1, B1, C1, D, E3, F1 and G; differences of <50% are in bold font.

**Table 5:** Amino acid differences among BoNT/A subtypes.

Subtype		Maximum Between-Subtype Differences (%)								Maximum Within-Subtype Difference (%)
		A1	A2	A3	A4	A5	A6	A7	A8	
A1	*n* = 80	-----	10.1	15.4	10.6	2.9	4.3	6.2	6.7	0.5
A2	*n* = 34		-----	7.0	11.7	9.7	8.3	10.3	6.6	2.5 *
A3	*n* = 4			-----	15.6	15.0	13.8	15.2	12.3	0.2
A4	*n* = 1				-----	12.6	12.2	13.3	10.9	-----
A5	*n* = 3					-----	4.2	5.6	6.6	0.2
A6	*n* = 2						-----	7.0	7.0	0.1
A7	*n* = 1							-----	8.7	-----
A8	*n* = 2								-----	0.1

* Within-subtype differences decrease to 0.8% after removal of toxin formed by CDC 2171.

**Table 6:** Amino acid differences among BoNT/B subtypes.

Subtype		Maximum Between-Subtype Differences (%)								Maximum Within-Subtype Difference (%)
		B1	B2	B3	B4	B5	B6	B7	B8	
B1	*n* = 18	-----	4.4	4.0	6.8	3.9	3.9	5.3	4.6	1.1
B2	*n* = 38		-----	**1.6**	6.1	4.7	**1.6**	4.2	4.2	2.9 *
B3	*n* = 1			-----	6.3	4.3	**1.9**	4.3	**2.5**	-----
B4	*n* = 16				-----	7.1	6.9	6.4	7.1	1.9
B5	*n* = 9					-----	4.6	5.7	5.4	0.8
B6	*n* = 6						-----	4.9	4.4	0.2
B7	*n* = 2							-----	5.6	0.1
B8	*n* = 1								-----	-----

* When 5 outliers are removed, the within-subtype difference decreases to 0.9%. Where differences are <2.6%, the percentage difference is shown in **bold**.

**Table 7:** Amino acid differences among BoNT/C and BoNT/D subtypes.

Subtype		Maximum Between-Subtype Differences (%)				Maximum Within-Subtype Difference (%)
		C1	CD	D	DC	
C1	*n* = 11	-----	24.2	48.8	35.3	0.1
CD	*n* = 8		-----	30.7	48.2	2.0 *
D	*n* = 2			-----	23.5	1.8
DC	*n* = 2				-----	0.1

* When 2 outliers are removed, the within-subtype difference decreases to 0.3%.

**Table 8:** Amino acid differences among BoNT/E subtypes.

Subtype		Maximum Between-Subtype Differences (%)												Maximum Within-Subtype Difference (%)
		E1	E2	E3	E4	E5	E6	E7	E8	E9	E10	E11	E12	
E1	*n* = 23	----	**0.9**	**1.8**	2.7	3.1	3.0	**2.1**	3.8	10.9	4.6	6.6	7.1	0.2
E2	*n* = 2		----	2.6	3.0	3.7	3.6	2.9	3.0	10.7	4.2	6.2	6.9	0.0
E3	*n* = 143			----	4.4	4.9	4.1	2.6	4.3	11.3	5.3	7.4	7.5	0.6
E4	*n* = 3				----	5.1	3.1	3.8	3.9	10.1	5.2	7.3	7.5	0.0
E5	*n* = 11					----	5.2	5.2	5.9	10.6	6.6	8.1	6.5	0.0
E6	*n* = 3						----	3.6	3.2	11.8	4.4	6.9	9.0	0.1
E7	*n* = 2							----	**1.7**	10.9	3.2	6.5	7.6	0.0
E8	*n* = 1								----	10.6	**2.1**	5.6	8.1	----
E9	*n* = 1									----	10.6	11.0	8.6	----
E10	*n* = 36										----	4.3	8.1	0.8
E11	*n* = 9											----	9.0	0.0
E12	*n* = 1												----	----

Where differences are <2.6%, the percentage difference is shown in **bold**.

**Table 9:** Amino acid differences among BoNT/F subtypes.

Subtype		Maximum Between-Subtype Differences (%)								Maximum Within-Subtype Difference (%)
		F1	F2	F3	F4	F5	F6	F7	F8	
F1	*n* = 10	----	16.6	16.1	7.8	30.2	12.6	26.3	3.7	0.1
F2	*n* = 4		----	3.0	16.5	26.0	10.2	31.4	16.9	0.3
F3	*n* = 2			----	16.2	26.0	10.2	31.1	16.5	0.1
F4	*n* = 11				----	30.6	13.1	28.1	7.4	0.6
F5	*n* = 7					----	26.4	36.2	30.9	0.1
F6	*n* = 7						----	30.2	13.1	0.2
F7	*n* = 9							----	28.0	1.7*
F8	*n* = 1								----	----

* Within-subtype differences decrease to 0.8% after removal of BoNT/F7 formed by ATCC43756.
